# Consenting futures: professional views on social, clinical and ethical aspects of information feedback to embryo donors in human embryonic stem cell research

**DOI:** 10.1258/ce.2009.009038

**Published:** 2010-06

**Authors:** Kathryn Ehrich, Clare Williams, Bobbie Farsides

**Affiliations:** * Centre for Biomedicine & Society, King's College London, Strand, London WC2R 2LS, UK; † Brighton & Sussex Medical School, Brighton, East Sussex BN1 9PX, UK

## Abstract

This paper reports from an ongoing multidisciplinary, ethnographic study that is exploring the views, values and practices (the ethical frameworks) drawn on by professional staff in assisted conception units and stem cell laboratories in relation to embryo donation for research purposes, particularly human embryonic stem cell (hESC) research, in the UK. We focus here on the connection between possible incidental findings and the circumstances in which embryos are donated for hESC research, and report some of the uncertainties and dilemmas of our staff participants. We explore the views of our study participants in relation to two themes: (1) rights to information and anticipating how donors might be informed about future research findings and (2) occupational work goals and trust.

## Introduction

The term incidental finding (IF) has been defined by Wolf *et al.*^1^ as ‘a finding concerning an individual research participant that has potential health or reproductive importance and is discovered in the course of conducting research but is beyond the aims of the study’ (p. 219). Examples for the purposes of this paper might be genetic information that may be produced as part of future research projects, or the discovery of viral contamination in a human embryonic stem cell (hESC) line that was not tested for (because not yet discovered when the embryos were first donated or the UK Stem Cell Bank [UKSCB] first received the stem cell line).

Communicating information from IFs back to the original donors of human biological material to research touches upon some of the most important conflicts in bioethics. These concern the right of individuals to control of their own medical information versus the usefulness of medical information on a larger scale (family, population groups, national and global);^2^ the right of individuals to such knowledge versus the right *not* to be informed;^3–5^ and the difficulty of adhering to the principle of clear and informed consent for research participants when researchers cannot predict or control many of the future implications of participation.^6–8^ These issues also bring into sharp relief the relationship between clinical and research ethics as a key aspect of the current emphasis on the ‘translation’ of science^9^ to the clinic and beyond.

This paper aims to provide empirical data on the views of assisted conception unit (ACU) and hESC research staff to contribute to the literature on social and ethical aspects of disclosure of IFs to research participants (in this case embryo donors) and promote discussion of the issues. We focus on the connection between the circumstances in which embryos are donated for hESC research in the UK and the possible future disclosure of IFs (concerning genetic, viral or bacteriological information) to embryo donors.

Following this introduction we set out the background to the particular case of IFs in hESC research in the UK. We then go on to report on this aspect of our ongoing multidisciplinary study on ethical frameworks for donation of embryos for hESC research in three linked ACUs and stem cell laboratories. Professional clinical and hESC research staff articulated some uncertainties, dilemmas and concerns about the policies and practices that are in place or may be needed regarding communication of IFs to embryo donors. After a description of the methods used in the study, we explore the views of our study participants in relation to two themes: (1) rights to information and anticipating how donors might be informed about future research findings and (2) occupational work goals and trust.

## Background

In the UK, the Human Embryology & Fertilisation Authority (HFEA) approves and licenses all studies on human embryos, and requires that when stem cell lines are grown from human embryos, a sample from those lines should be deposited in the UKSCB so that approved stem cell researchers from the UK and abroad can access them for further research. The embryos used for this research are those that patients cannot, or choose not to, use for their own treatment. The assessment of their likely viability is determined by embryologists in the ACU (see Note [a]). Embryos that are judged unsuitable for transfer to the woman's womb at three to five days after fertilization, or cryopreservation for a later ‘frozen embryo transfer’, may be discarded or donated to research, including hESC research. In the latter case, they may be donated to stem cell laboratories directly linked to the ACU where the donors have received treatment or to other laboratories. Consent for donation of embryos to hESC research therefore takes place in ACUs, after embryological assessment. Fulfilment of the requirements of proper consent from embryo donors may be the responsibility of nurses, clinicians or stem cell coordinators. An initial consent process is undertaken before treatment commences using a standard HFEA form. If patients are willing to consider donating embryos to research, a further process of consent to a specific research project takes place once the potential donors know if they have embryos that will not be used for their own treatment.

The UK national network of human embryonic stem cell coordinators (hESCCO – see Note [b]) drew attention to some potential dilemmas arising from the current regulations concerning feedback of information to donors of embryos for hESC research, and therefore problems for the donation consent process.^10^ The UKSCB Code of Practice (COP)^11^ *recommends* ‘that donors are informed that no individual feedback will be given on tests performed by the UKSCB or research results of subsequent studies unless in the unlikely event that the UK Stem Cell Bank Steering Committee (SCBSC) considers that donors should be contacted in relation to confirmed test results of direct relevance to the donor's, or the donor's family health’ (p. 17) (see Note [c]). In addition, the COP only requires that informed consent to donate embryos for hESC research should include confirmation of ‘*whether* any information emerging from tests done on the genetic material will be fed back to donors’ (p. 15, italics added). Further, the COP requires that donors must consent in writing *whether* they agree to be contacted in future. These stipulations could be interpreted as allowing researchers to rule out any further information feedback. However, that would be inconsistent with the SCBSC's option to re-contact donors in particular circumstances. In short, there appears to be room for confusion because the ultimate decision whether or not to contact donors lies with the SCBSC, yet the extent to which current consent forms ensure that donors would be able to choose to receive such information or *not* in the future is unclear. Bell and Devaney^12^ argue that such gaps and overlaps, and the lack of a comprehensive and cohesive regulatory system to govern stem cell research, pose a significant hindrance to the progress of stem cell science in the UK.

In 2008 the MRC/BBSRC Stem Cell Dialogue study^13^ reported that general acceptance (among members of the public, specialists and experts who participated in the study) of approaching assisted conception patients to donate embryos for hESC research hinges on assumptions that fully informed consent will be obtained, and that all clinical and scientific endeavours will be regulated effectively. However, ‘the difficulty of monitoring the ultimate use of stem cells lines created through embryos was acknowledged’ (p. 40), including the question of ‘whether stem cell banks should have to tell donors if they discovered diseases in their cells which would affect them later in life’ (p. 24). The report suggests that ethics committees will increasingly need to account for donor and public views as the science develops, and that ‘advances in the field were seen to precipitate major challenges for regulation and informed consent – particularly in terms of the development of different treatments from embryonic stem cell lines and governing the purposes to which research was put’ (p. 61), and in the context of global access (from countries that may have different applicable regulations) to UK banked stem cell lines. It is therefore important to try to ensure that current consent processes will allow future researchers using hESCs to have documentary evidence that the embryos have been ethically obtained. Yet difficulties experienced at the UKSCB in gaining adequate ‘paper trails’ have shown how challenging it can be to foresee the requirements for documentation that may be needed in the future.^14^

We focus on IFs in hESC research for several reasons. First, there has been substantial debate about making *intended and foreseeable* research results available to participants, yet there has been little research on how researchers should anticipate and manage the discovery of IFs in human subjects research^1^ and there are important differences between intended and incidental research findings. For example, IFs are discovered by researchers but the information may be beyond the aims of the original study. One consequence of this is that the researchers may not be certain how to interpret the findings, and they may need to refer to clinical colleagues to consider instigating clinical standard tests before deciding on how to deal with the implications of disclosure of information to donors. There is also a significant literature on disclosure of IFs following *clinical* investigations but less on disclosure following *research* results. For example, Lucassen and Parker^15^ discuss the closely related but only partially analagous ethical issues in relation to disclosure of misattributed paternity in *clinical* genetic family studies, but there is little on disclosure of IFs relating to genetic *research*, for example following genomic microarray analysis or large-scale genomic epidemiology research. There is a lack of research on the management of IFs in stem cell research, but there is evidence of incomplete consensus on policy and practice regarding IFs in research more generally. Wolf *et al.*'s^1^ recent multidisciplinary empirical and normative evaluation of the ethical, legal, scientific and clinical implications of IFs and how they have been handled in research in the US stresses the importance of achieving consensus on this issue, as the challenges are becoming more serious, particularly as genetic research technologies become more powerful and large-scale. Wolf *et al.* argue that there is a need for greater clarity among funding, academic and ethical review bodies as to the ethical duties of researchers regarding IFs in research, and Lawrenz and Sobotka's analysis^16^ of publicly available guidance and consent forms (mainly but not exclusively from the US context) concluded that there is ‘very little public guidance available for researchers as to how to deal with incidental findings… [and] the guidance available is not consistent’ (p. 255). Similarly, the international comparative analysis by Chadwick *et al.*^4^ of 27 policies on ethical guidelines for biobanks found a great diversity among existing guidelines on the topic of feedback to participants. Topics on which they found variation in guidance included whether investigators or biobanks should be obliged to inform research participants about IFs; whether participants should be informed directly, or through appropriate clinicians; whether anonymity should be complete or reversible; and the issue of participants' rights to know and *not* to know. It has been argued in the US context that there are increasing public expectations for the return of research results in various forms^17^ and Knoppers *et al.*^18^ claimed that principles for international ethics guidelines on disclosing, in certain circumstances, individual genetic research results to participants have now emerged: ‘At the international level, there now exists an ethical duty to return individual genetic results subject to the existence of proof of validity, significance and benefit… the right of the research participant not to know also has to be taken into consideration’ (p. 1170). The evidence that such principles are emerging is significant because of the national and international collaborative nature of hESC research and the need for scientific research to proceed across diverse jurisdictions, despite a lack of complete consensus or harmonized regulation.^19,20^

We conclude from these indications for concern that if embryo donors to hESC research, clinicians, scientists and ethics committees are to be confident that consent is sufficiently informed and effective within the UK, and consistent with the emerging international principles, there is a need for greater knowledge of how these issues affect clinical practice and consenting processes in particular. Our position as a multidisciplinary team engaging in empirical study of bioethical issues is to provide insight into the contexts in which consent to embryo donation for hESC research is taken, which is under-represented in the literature. In this paper we report the views of staff working in two UK ACUs and linked stem cell laboratories and interpret their comments from a social science perspective. Thus the paper responds to the call for multidisciplinary empirical work on bioethical issues,^21–23^ and in particular adds to the small but growing body of ethnographic research on the views and experiences of *staff* in these fields in the UK.^24–27^

## Methods

This paper reports on one aspect of an ongoing multidisciplinary, ethnographic study that is exploring the views, values and practices, or ethical frameworks, drawn on by professional staff in ACUs and stem cell laboratories in relation to embryo donation for research purposes, particularly for hESC research. Following national and local Research Ethics Committee approvals, the study methods include clinical and laboratory observation, interviews and ethics discussion groups (EDGs)^28^ with staff from three ACUs and linked stem cell laboratories in the UK. Staff disciplines include nursing, obstetrics and gynaecology, embryology, stem cell scientists, counselling, and clinical and science management. As a multidisciplinary team comprising three social scientists, an ethicist and a consultant embryologist/ACU director, we are exploring the social processes, meanings and institutions that frame and produce ‘ethics’ and ‘ethical problems’ in these settings.

The study sites are three ACUs in teaching hospitals in England, which offer a mixture of National Health Service (NHS), privately or ‘self-funded’ NHS treatment, and three stem cell laboratories at the associated universities. The clinics provide a range of services including *in vitro* fertilization (IVF) to women and couples who need fertility treatment. Participants from across a broad range of disciplines are being recruited by group introductions to the study, followed by individual approaches from the main researcher. The interviews are being conducted by the main researcher (KE) as ‘guided conversations’,^29^ lasting between one and two hours. Open-ended questions and an informal interview schedule are used, with topics such as the ethical differences between donation of embryos for treatment and research, the details of how and when consents are taken, how embryos are selected for treatment and views on the regulation of clinical and scientific aspects of reproductive technologies.

This paper draws on 25 staff interviews and two EDGs held at two of our study sites between February 2008 and February 2009. For our analysis of the transcripts we used a modified version of the framework approach^30^ following close readings of the transcripts. Emerging themes from the study as a whole, including those presented here, were discussed in the team meetings. While we do not claim that the quotes we present here are statistically representative of the views of staff in our sites, or in the UK generally, they are indicative of common themes that arose from the fieldwork at these two sites, and quotes used in this paper are intended to illustrate topics identified in the interviews and EDGs. Note that for this paper we use the word ‘donors’ when referring to women and couples in the context of their possible or actual donation of embryos for research, and ‘patients’ when staff have used this term. Also, we use the term ‘participants’ and broad occupational categories (with study numbers) to describe the members of staff who engaged in interviews and EDGs. We acknowledge that for all of these practices some readers might prefer other conventions.

## Results

Although participants agreed on many local and national policies relating to embryo donation and information provision in principle, they also expressed some uncertainties based on their clinical or scientific experience that did not seem to align with these policies, and commented on some unresolved dilemmas concerning the contemporary and long-term implications of IFs in hESC research. These included considerations about communicating information arising from IFs to embryo donors; demarcation of occupational roles pertinent to consent processes; and how these issues might ‘fold back’ into current clinical and scientific practices in anticipation of some of the future possible scenarios envisaged.

Our discussion of these data is organized into two themes. The first theme relates to embryo donors' rights to being informed about IFs and how this information would be communicated to donors. The second theme illustrates how participants in our study perceived differences in occupational work goals between clinical and research staff, and explores the significance of these for determining who should take consents for donation of embryos to hESC research, and who should communicate possible future IFs to embryo donors.

### Rights to information and anticipating how donors might be informed about future research findings

As discussed earlier, the details of how donors might be informed about serious medical issues which come to light in the context of research with human embryos and hESC lines are not set out in the HFEA COP (2006)^31^ or UKSCB COP (2006).^12^ Participants commented on their concerns about some of the ethical and practical implications of this, referring to broad concepts about patients' rights.

Embryologist 7 was unsure how information from IFs would be communicated to donors. She weighed up how it might seem easier just to say that all data will be irreversibly anonymised against questions about patients' rights to information, and rights not to know, and the dilemmas this would cause in practice:

‘You know, you can't phone them up and say, “Oh do you want to know about your stem cell line?” Because then they'll go, “Well why are you phoning?”’.

She reflected on the pros and cons of receiving unanticipated information, how it could affect a person's quality of life, their ability to get life insurance and then again considered whether it would be better simply to be treated if one becomes ill or be able to have the option of preventive treatments:

‘Probably the simplest thing would be just to say that everything is anonymised and there's no feedback. But then… do people have the right to know?’

Several other participants expressed similar wavering thoughts: whether embryo donors should or should not receive any further information; awareness that the current guidelines could override such a wish; and the situation that donors are not asked what their preferences would be.

A somewhat analogous scenario mentioned by some participants is that of informing ‘altruistic’ sperm donors (rather than the male partner in a treatment cycle) of the results of standard tests for sperm count and viral infection. Participant 18, whose role involves research management in the ACU and the stem cell laboratory, wished to avoid worrying patients unduly, but also thought that it is generally best to give people information. For sperm donation, donors have to agree before giving the sample how much information they want to receive, but this did not always resolve the dilemmas:

‘You see young men come in… and you see their sample, and you look at it and instantaneously, you know, “You're not going to have children naturally, or at least nigh on impossible”. And you have that twinge inside you that says, “Right, you have to know this”. And when [they have indicated on the form] that they don't want to know anything… you're in that horrible position of saying, “I really want to tell you, but I can't”.’

Many of our research participants raised similar questions about how to look ahead, at the point of donor consent, to some of the decision-making processes that might arise in the future. However it was difficult to decide on the best way of anticipating some of these difficulties. Scientist 9 was in some respects against the idea of going back to the donors with information, for example to tell them if an hESC line had been successfully derived from their embryo. He thought this could be positive or negative, but most probably would be negative for women who wanted to move on from treatment.

Scientist 19 thought that it would be good for people to be able to specify particular preferences about further types of knowledge that they would or would not welcome:

‘It's all about giving people the freedom to do and give what they want, and then them feeling like they have been in control of that process, rather than them being steamrollered and then coming out of it feeling a bit mangled and thinking, “All these people have just done this to me”.’

The range of views and uncertainties in this theme, including the ideas staff expressed about patients'/donors' rights to information, and how this might be offered back to donors, particularly if this might occur after a considerable lapse of time, was found across participants in all disciplines. Aware that such information could be either helpful or unwanted, they could foresee potential dilemmas because there are no arrangements currently in place to allow donors to control the process themselves, other than refusal to donate. They were unclear about how disclosure of information could best be handled without knowing beforehand how individual donors would want to be approached.

In the face of these dilemmas about how patients/donors could be approached and informed about some possible implications of embryo donation to hESC research, many staff could not resolve all of the questions they raised yet had to find ways of acting within the ethical expectations and standards of their professional roles. We intend to discuss in more detail the relationship between knowledge, control and voluntariness as requirements of informed consent for *donors* in this context elsewhere. However, here we have shown how the long-term and uncertain nature of hESC research presents clinical staff with dilemmas because they cannot provide information about all the future research possibilities that may apply to hESC research, and this means donors' knowledge and control is limited. Key questions for staff involved with obtaining consents from donors are therefore, ‘How can consent be fully informed if the future uses of the tissues are not known? What rights do embryo donors have to information that emerges in research involving stem cells using their embryos? Should donors have more control over whether (or not) they wish to receive such information?’

### Occupational work goals, orientations and trust

For some staff, one resolution of the dilemmas discussed in the preceding section was to draw on their belief that as professionals with work goals and orientations that value and respect patients' main goal of achieving pregnancy, they could be trusted to act in patients' best interests. This resolution is vulnerable to the ongoing difficulties of interpreting best interest in particular cases, and therefore raises another key question: ‘Who can know what is in the patient's best interest in such circumstances?’ In this section we focus on the ‘who’ in that question, and discuss views from both clinical and research staff relating to occupational work goals, orientations and trust.

It should be noted here that in these two sites and up to the time we had completed the 25 interviews analysed for this paper, there were no appointed stem cell coordinators for a number of reasons, for example the clinics were not at that time approaching couples to donate embryos specifically to an hESC research project, although they had in the past and envisaged doing so again. Nurses in the two ACUs reported from here were responsible for discussing the HFEA consent forms couples are required to consider before commencing treatment. These forms require patients to state their preferences about donating embryos to research in general. Some clinical staff (i.e. nurses, doctors and embryologists) thought that research staff (i.e. scientists and research managers from the hESC research laboratory) would be responsible for providing further information for specific research projects and obtaining separate consent at a later stage, usually (in these two clinics and during this period of the study) when embryos had been cryopreserved and couples decided they no longer wished to store them for their own treatment. However, there were diverse views among clinical and research staff about the principle of separation between clinical and scientific research interests (see Note [d]). Some clinical staff agreed that researchers should ‘take consents’ (see Note [e]) on the basis that clinical staff could not answer all the questions that patients might have. For example, Nurse 25 said:

‘It should be someone doing the research mainly because… they're the experts really. And the questions that you might get asked… it's all sort of specific and I wouldn't know it all really’.

However, other clinical staff disagreed with this policy and thought that clinical staff were the best people to discuss research with potential donors because they believed their work orientations were more ‘patient focused’, for example helping patients achieve pregnancy. For example, Embryologist 7 argued:

‘I have issues with [stem cell researchers obtaining consents] because I think their job depends on getting embryos for research…I think there's a potential to pressurise patients… it's better almost [for] the person doing the treatment to ask them to donate to research because if I'm doing the consent to research, my primary objective is to get that patient pregnant… I don't think I am pressurising that patient at all into research. My focus is always on treatment, not research’.

Claims that clinical staff were more likely to have a ‘patient focus’ could also lead to further claims about patients' trust in clinical staff. Nurse 1 argued that the treatment work goals in the clinic, in contrast to research in the laboratory, meant that patients could put their trust in the clinic staff. Most patients did consent to donating embryos for research that they could not use for their own treatment, and had consented to donate embryos to stem cell research in the past as well:

‘That's because they trust us. And therefore we've got to keep our end of that right and make sure that we are doing our best to get them pregnant because that's what we're about really…if you're a researcher, then you've got a different agenda, haven't you?’

A similar statement was made by Clinician 8. After setting out a clear picture of how couples would be approached for donation to research by staff who are ‘separate from anything to do with clinical practice’, the clinician went on to describe the perspective of patients who are interested in donating embryos for research:

‘They see it as the unit that's done its best to help them have children, and who you hope therefore they feel quite good about, endorses the decision to suggest donating for stem cell research.’

These statements from the nurse and clinician are interesting because they could be taken to imply that although the different ‘agenda’ of researchers might stand in contrast with that of the clinic, at the same time trust engendered in the clinical relationship could be seen as *facilitating* patients' agreement to donation for research.

It is also important to note that at the time these statements were made, most embryos eligible for donation to research (in these units, at the time of our interviews) had been in cryopreservation storage. It could be argued that the passage of time since the couple were in active treatment could mean relationships of trust with clinical staff would be harder to claim. However, the clinical staff making such statements had been employed in the ACUs for a considerable length of time so might be expected to be recognized by patients. They might also contend that their clinical orientation, direct contact with patients, or ‘patient focus’ formed the basis of the trust relationship rather than long-term contact. Embryologist 2 illustrated this view:

‘Although the stem cell team as a whole would understand the ethical issues behind using human embryos and gametes, I think sometimes they're a little bit removed from the patients to understand that, you know, it's [the patients'] kind of future babies, possible future babies, that you're using for research… it's quite a delicate issue.’

Referring to ‘possible future babies’ in this way reflects a patient focus because some patients do think and talk about their embryos as ‘babies’, whereas stem cell lab researchers talk about dealing with embryos and stem cells, but not ‘babies’. The embryologist has also pointed out that researchers are ‘removed’ from patients, so despite understanding the ethical issues, they cannot build up the kind of trust relationship clinical staff are more able to do through direct contact with patients.

However, if research staff also expressed professional values that upheld the principle of separation between clinical treatment and research in the best interests of the patient, this was not necessarily an indication of prioritizing their research over patients' treatment. Scientist 17, for example, said:

‘Strictly speaking, I'm independent from all of that [treatment], so the idea is that in order to avoid coercion of patients, then the scientists don't get involved with the patients. …[However] although I'm interested in research, I actually personally would rather that people got pregnant than me have an embryo. So I don't feel that I would necessarily be particularly pushing them to do that, but I guess if you're going to make a rule, then it's much safer and cleaner to have scientists as a separate thing’.

Another reason this scientist gave for not feeling in the best position to take consents for donation to stem cell research was that as a scientist, s/he did not have training for direct contact with patients.

Comments from our participants about different work goals and uncertainties about possible future information feedback suggest that the separation of work processes and lack of full understanding of what might happen in the future with stem cells derived from donors' embryos combined to create some concerns. Day-to-day pressure of work did not allow clinical staff sufficient time to become fully informed about the future implications of embryo donation, and lack of regular contact between the teams exacerbated uncertainties about each other's current and future responsibilities. The opportunity to meet in mixed discipline EDGs was appreciated for this reason (ascertained in the post-EDG evaluation exercise), as colleagues could learn about and discuss practices and policies across the different staff groups.

In Fisher's^32^ study of how ethics are constituted ‘on the ground’ in pharmaceutical clinical trials, similar concerns about the separation of research and clinical relationships were raised by research coordinators who experienced role conflicts and potentially divided obligations to patients and to pharmaceutical companies, and such conflicts led to staff developing informal ethical practices in an attempt to ‘reinsert care into research’ (p. 689). A similar example of this everyday constitution of ethics could be seen in our data, when many staff (both clinical and research) took the position that if patients were ‘uncomfortable’ with any possible aspect of donation for research, including not being able to know what some of the implications might be, then it was simpler but also ethically preferable to advise them not to donate. The idea of patients and staff being ‘comfortable’ about particular processes (aspects of consent for example) was often referred to and seemed to operate as a rule of thumb when explaining their ethical positions. In these circumstances it could be argued that advising patients not to donate solved both staff and patients' problem of not being ‘comfortable’, and allowed some staff to feel confident that prioritizing clinical care over research meant patients could trust them.

## Discussion

There has to our knowledge been no social science investigation of the actual practices and policies for managing possible communication to embryo donors of IFs that might arise from hESC research, and the consequent ethical dilemmas that may be involved for clinical and scientific staff, and this paper aims to address that gap.

However, there is an emerging body of literature on the views of various stakeholders in the UK on ethical and social issues relating to stem cell science. For example, Franklin and Robert's^27^ ethnographic work in this field has documented the processes by which ethical quality control, so essential to the future use of embryos in the derivation of hESCs, centres on transferring gametes and embryos from assisted reproduction settings to stem cell laboratories. Plans for long-term and heavy UK investment in hESC research emphasize its promise for future utility in healing and cure,^33^ and bioethical provenance has become an essential tool in the international brokerage of hESC research.^34^ Yet Michael *et al.*^35^ argue that stem cell scientists themselves have articulated vague and opaque futures that perform uncertainty and wariness in the present. Our data suggest that this may have some impact on ethical, clinical and social aspects of embryo donation as staff seek to resolve the day-to-day dilemmas linked with these uncertainties.

Roberts and Throsby^36^ and Hallowell *et al.*^37^ point out ethical concerns stemming from the elision between research and clinical care and the principle of separating these two functions. Roberts and Throsby^36^ address this aspect of a UK research programme that offers women undergoing IVF reduced fees for donating eggs to *research* (as distinct from existing schemes for egg ‘sharing’ for other couples' *treatment*). They argue that ‘the research nurse is positioned as a buffer between the patient and the clinic’, but ‘the separation between the [research] nurse and the clinical team is hard to sustain, particularly for patients for whom nurses and clinicians remain inter-connected actors within the clinical setting’ (p. 165). Hallowell *et al.*^37^ studied a similar ‘demarcation problem’ in clinical genetics, where ‘patients find it difficult to differentiate genetic testing undertaken in research protocols from that offered as part of their clinical management’ (p. 1) and argue that this is particularly important in relation to consent processes. They point to increasing evidence that participants in genetic epidemiological studies ‘expect to receive personal feedback about their genetic risk status… even when it has been explicitly stated that such information will not be forthcoming’ (p. 2), a point echoed in the exploration by Dixon-Woods *et al.*^38^ of the ‘therapeutic misconception’ in consent to research participation. For this reason, many of Hallowell *et al.*'s^37^ research participants described means of avoiding this problem by using the concepts of space and time to maintain boundaries between their clinical and research activities. Our data on the separation between clinical care and hESC research also point to the use of space and time to maintain boundaries, but also to lay claim to the trust of patients. For example, many participants in our study (at the time of the interviews) assumed that (frozen) embryos would only be recruited at a later date after treatment had finished, so time separated research from clinical treatment. Everyday concerns about the separation of clinical and research functions and possible re-contact of donors about IFs in the future could be resolved to some extent by resorting to a further assumption that responsibility to handle decisions about re-contacting donors would arise *elsewhere* from *future* discoveries (e.g. researchers rather than clinical staff, researchers in other places, regulatory bodies or ethics committees). Thus some staff use spatial and temporal concepts to demarcate the future and other occupational groups as spaces in which resolution of possible conflicts might occur outside of the boundaries of immediate clinical practice. This helps to resolve, for example, what might be thought of as the intrusion of having to consider donation consent process and dilemmas such as those discussed above regarding IFs from hESC research into clinical relationships with ACU patients.

Stephens *et al.*'s^14^ detailed ethnography of the UKSCB has illuminated how reliance on developing (professional) social networks of trust to establish the ethical provenance of stem cell lines became necessary because of the previously unforeseen need for otherwise absent documentary evidence. Detailed scrutiny of applications to bank lines in the future or from overseas seems (according to their research) less, not more, likely, which suggests that reliance on trust networks might also increase. In some ways the importance of similar networks of trust can be seen from our participants' accounts of negotiating between different clinical and research goals and responsibilities and forming trust relationships with patients/donors; and staff reliance on ethics committees and regulatory bodies to anticipate possible future contact with donors in a way that those currently taking consents would find acceptable. However, another way of reading this might be as an alert to members of ethics committees whose role includes scrutinizing the accountability of research, and consent in particular, with an eye to how disclosure of future discovery of IFs might be facilitated by contemporary consent agreements.

Pertinent to this is that some of our research participants expressed concerns about donors of embryos for research lacking an opportunity to indicate whether they would *not* wish to be given information, which supports the attention drawn to this issue by the hESCCO group. This point offers some support for Manson and O'Neill's^39^ critique of standard models of informed consent in biomedical ethics, in which they argue that regulators' narrow focus on *types* of information to be disclosed by those who seek consent and on decision-making in consent processes addresses only part of the problem, and offers inadequate solutions to the kinds of concern and uncertainty our participants raised. Some of their concerns arise because the current codes of practice applicable in the UK leave open possibilities for short- and long-term problems relating to donor consent and potential future disclosure. For example, in addition to the lack of clear protection of what some donors (and staff) may regard as a right *not* to be informed, the guidelines do not suggest *how* researchers could ensure communication about such information is sufficient or effective; there is little detail for clinical and scientific staff taking consents from donors as to how IFs would be handled in practice once the SCBSC decides that donors should be informed. Consequently, ethics committees considering research protocols may find it difficult to envisage how effective forms of accountability will take place in practice. Manson and O'Neill^39^ propose new ways of thinking about consent in terms of communicative transactions to achieve intelligently placed trust or refusal, and our data suggest that both clinical and research staff are concerned that patients/donors should be able to place their trust in staff since there is such a high degree of uncertainty about possible future developments in the field of hESC research and therefore the exact uses of the stem cells created from their embryos.

As these arrangements in regard to hESC research are new and there is incomplete consensus or guidance on the practical details of how information might be offered back to donors, we think it premature to offer normative comment at this stage. However, our observations and analysis of interview and EDG data provide evidence of clinical and research staff formulating and engaging with important, ongoing issues:

- Obtaining fully informed consent in the absence of certain knowledge regarding the future uses of tissues;
- Ascertaining the right of donors to information which emerges in the course of research which relies on their donated embryos;
- The extent to which donors can exercise control and choice over whether or not they wish to receive such information;
- The establishment of who possesses expertise when judging the best interests of the donors.

We conclude that even if there are few IFs in hESC research that are referred to the UKSCB Steering Committee, these questions have an impact on how consent processes in these circumstances can be fully informed, and therefore on the quality of consenting processes for thousands of assisted conception and preimplantation genetic diagnosis patients. The possible future impact of this for the ethical provenance of stem cell lines, the progress of research and communications with embryo donors has yet to be seen, but there is some evidence that achieving greater consensus on communication of IFs from research more generally is regarded as urgent. We believe that the effort towards consensus and clarity will be aided by further empirical research on how staff in research laboratories, clinical units and research ethics committees, as well as professional and lay members of national policy-making and regulatory bodies have addressed the discovery and management of IFs by hESC researchers, donor views and experiences, emerging policies on possible future disclosure and the right not to be informed, and to document instances of anticipation and management of IFs that arise. Tracing the social, clinical and ethical dilemmas for these parties would contribute to empirical engagement with the challenges these pose to a wider set of stakeholders than we have been able to include in this study; and to the theoretical consideration of continuing debates on informed consent in this setting.

## Notes

[a] More detailed description of this process is not possible within the space available for this paper: please see Svendsen and Koch^40^ and Ehrich *et al.*^25^ for descriptions of ACU clinical decision-making and determination of what is a ‘spare’ embryo, and Cutting *et al.*^41^ for detailed information on a proposed embryological grading system for the selection of embryos for treatment. Further information about the regulatory arrangements for donation of embryos for stem cell research and depositing stem cell lines in the UK Stem Cell Bank can be found at www.hfea.gov.uk and www.UKstemcellbank.org.uk.

[b] hESCCO was founded in 2004, with the aim of enhancing cooperation between hESC derivation centres and assisted conception units. Stem cell coordinators from several centres, together with stem cell scientists, clinicians, embryologists and social scientists, met twice a year to discuss, *inter alia*, the considerable challenges in developing a national protocol for patient information and consent for embryo donation to stem cell research. In the two sites reported from here, and at the time of these interviews, there were no stem cell coordinators in post as envisaged by hESCCO.

[c] At the time of writing, the SCBSC COP^11^ refers to the stipulations of the 2004 EU Tissue Directive^42^ that embryo donors should also be traceable in the event of a *public health* concern, although the Directive itself is only concerned with stem cell derivation for *therapeutic* application and not *in vitro* research. Further, the COP notes that: ‘For hES cell lines derived in research grade facilities clinical use is unlikely. However, should such cell lines have unparalleled therapeutic potential, regulatory approval for clinical use may exceptionally be considered after detailed risk/benefit analysis’ (p. 16). This leaves open the question of whether in that case the Directive stipulations in regard to contacting embryo donors in the event of a public health issue would then apply, but we put that question aside for the purposes of this paper.

[d] Clinician EFED8 traced the influence of the Polkinghorne guidelines on thinking through ethical implications and guidelines for obtaining IVF embryos for hESC research; however Pfeffer^43^ points out that these were drawn up in relation to aborted fetuses and not other types of tissue.

[e] ‘Doing’ or ‘taking’ consents were phrases all staff used to refer to the process of explaining research projects to patients and obtaining written consent from them to donate embryos not used for treatment.
